# Repeatability of tumour perfusion measurement with [^15^O]H_2_O PET in prostate cancer

**DOI:** 10.1186/s13550-026-01375-2

**Published:** 2026-01-08

**Authors:** Mads Ryø Jochumsen, Jens Sörensen, Nana Louise Christensen, Margit Haislund, Michael Borre, Kirsten Bouchelouche, Lars Poulsen Tolbod

**Affiliations:** 1 Department of Nuclear Medicine, Aarhus University Hospital, Palle Juul-Jensens Boulevard 165, 8200 Aarhus, Denmark; 2 Department of Clinical Medicine, Aarhus University, Aarhus, Denmark; 3 Department of Surgical Sciences, Uppsala University Hospital, Uppsala, Sweden; 4 Department of Urology, Aarhus University Hospital, Aarhus, Denmark

**Keywords:** Tumour blood flow, Perfusion, Prostate cancer, [^15^O]H_2_O, Test-retest, Repeatability, Sample size calculation

## Abstract

**Background:**

Tumour perfusion is a universal cancer biomarker with potential value in characterising primary prostate tumours and longitudinal measurements for evaluation of treatment response. To evaluate whether a change in perfusion is significant, the reproducibility of the measurement must be known. [^15^O]H_2_O positron emission tomography (PET) is the gold standard for non-invasive quantitative perfusion imaging, however repeatability data on prostate cancer are currently unavailable. Hence, the aim of the present study is to determine the repeatability of [^15^O]H_2_O tumour perfusion in prostate cancer.

**Results:**

Thirteen well-defined MRI PI-RADS lesions from ten patients were studied. The repeatability of [^15^O]H_2_O K_1_ was 30% using both parametric image calculation and volume of interest (VOI)-based analysis. Intraclass correlation coefficient (ICC) was 0.89 and 0.91 for parametric image calculation and VOI-based analysis, respectively. A study sample size of 10 patients should be sufficient for detecting a relative change of 20% over time.

**Conclusions:**

[^15^O]H_2_O tumour perfusion in localised prostate cancer can be measured with a high repeatability, showing comparable performance when using parametric K_1_ perfusion maps and VOI-based analysis. For longitudinal evaluation, changes above 30% are likely to represent actual changes in tumour perfusion, for example as response to a specific treatment.

**Supplementary Information:**

The online version contains supplementary material available at 10.1186/s13550-026-01375-2.

## Background

Tumour perfusion is a universal cancer biomarker [1], with potential value in characterising the aggressiveness of primary prostate tumours and separating significant prostate cancer from insignificant cancer [2–5]. [^15^O]H_2_O positron emission tomography (PET) is the gold standard for non-invasive quantitative perfusion imaging. [^15^O]H_2_O PET has been studied as a tool for assessing treatment response across multiple cancer types [6–9]. In locally advanced breast cancer, change in tumour perfusion following neoadjuvant chemotherapy appear promising as a marker of long-term treatment effect and clinical outcome [10, 11]. Hence, a potential application for tumour perfusion imaging could be for evaluation of treatment response through serial measurements before, during and after a specific treatment [12]. To evaluate whether a change in perfusion is significant or not, the reproducibility of the measurement must be known. Previous test-retest studies found a high reproducibility of perfusion measurements with [^15^O]H_2_O PET in various non-prostatic tumours [13–15]. Previously, our group assessed the repeatability of perfusion measurements in primary prostate cancer using ^82^Rb PET [16], however repeatability data on [^15^O]H_2_O PET in prostate cancer are currently unavailable. Hence, the aim of the present study is to determine the repeatability of [^15^O]H_2_O tumour perfusion in prostate cancer.

## Methods

### Patient population

Ten patients with localised prostate cancer were recruited immediately before radical prostatectomy. All patients were biopsied and underwent radical prostatectomy. Gleason score and International Society of Urological Pathology (ISUP) grade were available from both biopsy and prostatectomy materials. All patients had a prior clinical prostate multiparametric magnetic resonance imaging (MRI) performed and histologically verified prostate cancer. Patients were excluded if they had hip alloplastic material or contraindications for MRI scan such as magnetic metallic implants, claustrophobia etc., and patients should be able to lie in the scanner for the extended scan duration.

### Imaging

All patients underwent two scan sessions, each consisting of a 5-minute dynamic pelvic [^15^O]H_2_O PET scan and a dynamic [^15^O]H_2_O PET heart scan for obtaining an image-derived input function. All scans were performed on a 3 Tesla GE Signa PET/MRI Quant Works scanner (GE Healthcare, Waukesha, Wisconsin, USA).

A standardised 400 MBq [^15^O]H_2_O bolus was injected via an automatic injection pump at the beginning of each bed position, followed by 5 min dynamic PET acquisition.

The scans were performed with a frame structure of 1 × 10 s, 1 × 5 s, 15 × 3 s, 5 × 5 s, 2 × 10 s, 5 × 15 s, and 4 × 30s. The voxel size was 2.8 × 2.8 × 2.8 mm3. Images were reconstructed using the VuePoint FX SharpIR reconstruction algorithm, which applies ordered subset expectation maximisation (OSEM) with point-spread-function (PSF) modelling and time-of-flight (ToF). The images were filtered with a 3 mm 2D transaxial Gaussian filter and a 3-point convolution filter [1:6:1] along the z-axis.

### Image analysis

Tumour volumes of interest (VOIs) of thirteen well-defined MRI PI-RADS lesions from ten patients were drawn directly on the T2 weighted MRI sequence by visual guidance, taking all available sequences into account. All VOIs were drawn using Hermes Affinity viewer version 3.0.1 (Hermes Medical Solutions, Stockholm, Sweden). Before further analysis, the T2 weighted MRI and parametric [^15^O]H_2_O PET K_1_ images were visually checked thoroughly for misalignment and alignment of the VOIs were corrected when necessary.

Tumour VOIs were subsequently transferred to the parametric [^15^O]H_2_O PET K_1_ images and [^15^O]H_2_O PET dynamic image series for extraction of the mean voxel tumour perfusion and extraction of tumour time-activity-curves (TACs), respectively.

Blood input functions for kinetic analysis were extracted automatically from the separate dynamic [^15^O]H_2_O cardiac scan series by cluster analysis to identify arterial clusters [17]. This heart image derived input function (HIDIF) was either used as it is or corrected for both delay and dispersion using the image-derived input function from pelvic arteries [5] (Table 1). The delay and dispersion corrected input function was denoted PIDIF.

Parametric K_1_ images were constructed using either PIDIF or HIDIF with voxel wise delay calculated from the Leading-Edge method [18]. Analysis on VOI-extracted TACs was similarly performed using both HIDIF and PIDIF, in both cases, including delay as a model parameter. The nomenclature is summarized in Table 1.

[^15^O]H_2_O wash-in (K_1_) (mL/min/mL) and wash-out (k_2_) (mL/min/mL) was calculated using a single-tissue compartment model. Kinetic analyses and blood input function extractions were performed using the aQuant Research Package (MedTrace, Hørsholm, Denmark).

**Table 1:** Nomenclature and corresponding modelling approaches used in this study

**VOI_PIDIF**	Model fit to mean TAC from VOI, including delay Input is HIDIF with delay and dispersion correction from PIDIF
**VOI_HIDIF**	Model fit to mean TAC from VOI, including delay Input is HIDIF
**Param_PIDIF**	Mean of parametric voxel values in VOI Input is HIDIF with delay and dispersion correction from PIDIF
**Param_LE**	Mean of parametric voxel values in VOI Input is HIDIF with voxel wise delay calculated from the Leading-Edge method

VOI = volume of interest, PIDIF = input function from pelvic arteries, HIDIF = heart image-derived input functions, Param = parametric, LE = leading edge, TAC = time-activity-curve

### Statistical analysis

The data were tested for normality using Shapiro–Wilk test. Based on Bland-Altman plots the variation between measurements do not seem to be depending on the average. The repeatability of the method was calculated by the method described by Bland and Altman [19] on log-transformed data. The within-patient/within-lesion coefficient of variance, repeatability, and intraclass coefficients (ICCs) were calculated for K_1_ and k_2_. We used the same statistical parameters and formulas as described in detail in Lodge et al. [14].

Sample size calculations for designing potential future studies were performed to be able to detect relative changes in tumour perfusion (K_1_) of −20%, −30%, and − 50% between 2 time-points in a longitudinal study. Calculations were performed for the 4 modelling approaches studied, using a 2-sided significance test of no difference for paired log-normally distributed data with a significance level of 5% and a power of 95%. Sample size calculations have been performed on log-transformed data.

Study data were collected and managed using REDCap (Vanderbilt University Medical Centre, Nashville, Tennessee, USA) electronic data capture tools, hosted at Aarhus University [20].

Statistical analysis was performed in MATLAB (MATLAB, MathWorks, Natick, MA) and Stata version 15.1 (StataCorp LLC, College Station, Texas, USA).

## Results

Patient characteristics are presented in Table 2, while test and retest measurements using different modelling approaches are found in Table 3.

**Table 2:** Patient characteristics. Three patients ([2, 4] and [7]) had two PI-RADS lesions in which tumour perfusion was assessed

Patient	Age	PSA	ISUP Grade (Prostatectomy)	ISUP Grade (lesion)	Lesion Size (cm^3^)	PI-RADS	Zone
1	60	8.2	1	1	2.7	4	TZ
2	64	17.2	3	1	3.0	4	PZ
				1	0.9	4	PZ
3	64	5.7	4	4	1.2	4	PZ
4	69	3.7	2	2	1.6	5	PZ
				1	1.3	5	PZ
5	59	16.0	2	1	2.9	5	TZ
6	65	18.3	2	2	3.2	5	PZ
7	58	7.6	2	1	0.73	4	PZ
				1	0.54	4	PZ
8	68	10.7	2	2	0.83	4	PZ
9	75	10.4	5	3	4.2	4	PZ/TZ
10	72	12.6	2	1	2.7	4	TZ
Mean	65.4 ± 5.62	11.04 ± 4.95			1.98 ± 1.18		
Median			2 [1; 5]	1 [1; 4]		4 [4; 5]	

Summary statistics are given as mean ± standard deviation for normally distributed continuous variables (Shapiro–Wilk test for normality for Age, PSA and Lesion Size with p-values > 0.05) and median with range for ordinal variables

**Table 3:** Test and retest tumour perfusion measurements (K_1_) using different modelling approaches

Patient	Tumour Size (cm^3^)	Test (K_1_)				Retest (K_1_)			
		VOI_PIDIF	VOI_HIDIF	Param_PIDIF	Param_LE	VOI_PIDIF	VOI_HIDIF	Param_PIDIF	Param_LE
1	2.7	0.073	0.075	0.094	0.083	0.096	0.095	0.119	0.112
2	3.0	0.208	0.198	0.231	0.197	0.201	0.184	0.227	0.206
	0.9	0.168	0.165	0.190	0.176	0.197	0.189	0.217	0.216
3	1.2	0.331	0.305	0.343	0.254	0.239	0.259	0.263	0.280
4	1.6	0.219	0.243	0.234	0.206	0.257	0.256	0.257	0.232
	1.3	0.212	0.247	0.234	0.197	0.214	0.213	0.217	0.236
5	2.9	0.328	0.327	0.357	0.328	0.305	0.275	0.310	0.229
6	3.2	0.237	0.235	0.263	0.201	0.201	0.188	0.220	0.185
7	0.73	0.204	0.204	0.247	0.240	0.190	0.197	0.232	0.234
	0.54	0.158	0.157	0.170	0.177	0.157	0.158	0.183	0.197
8	0.83	0.315	0.290	0.308	0.283	0.263	0.266	0.268	0.270
9	4.2	0.236	0.236	0.241	0.266	0.220	0.205	0.232	0.216
10	2.7	0.255	0.242	0.273	0.250	0.297	0.290	0.310	0.299
Mean *± SD*	1.98 ± 1.18	0.226 ± 0.072	0.225 ± 0.067	0.245 ± 0.070	0.220 ± 0.061	0.218 ± 0.056	0.213 ± 0.055	0.235 ± 0.051	0.224 ± 0.047

Summary statistics are given as mean ± standard deviation for normally distributed continuous variables (Shapiro–Wilk test for normality for all parameters with p-values > 0.05)

**Fig. 1:**
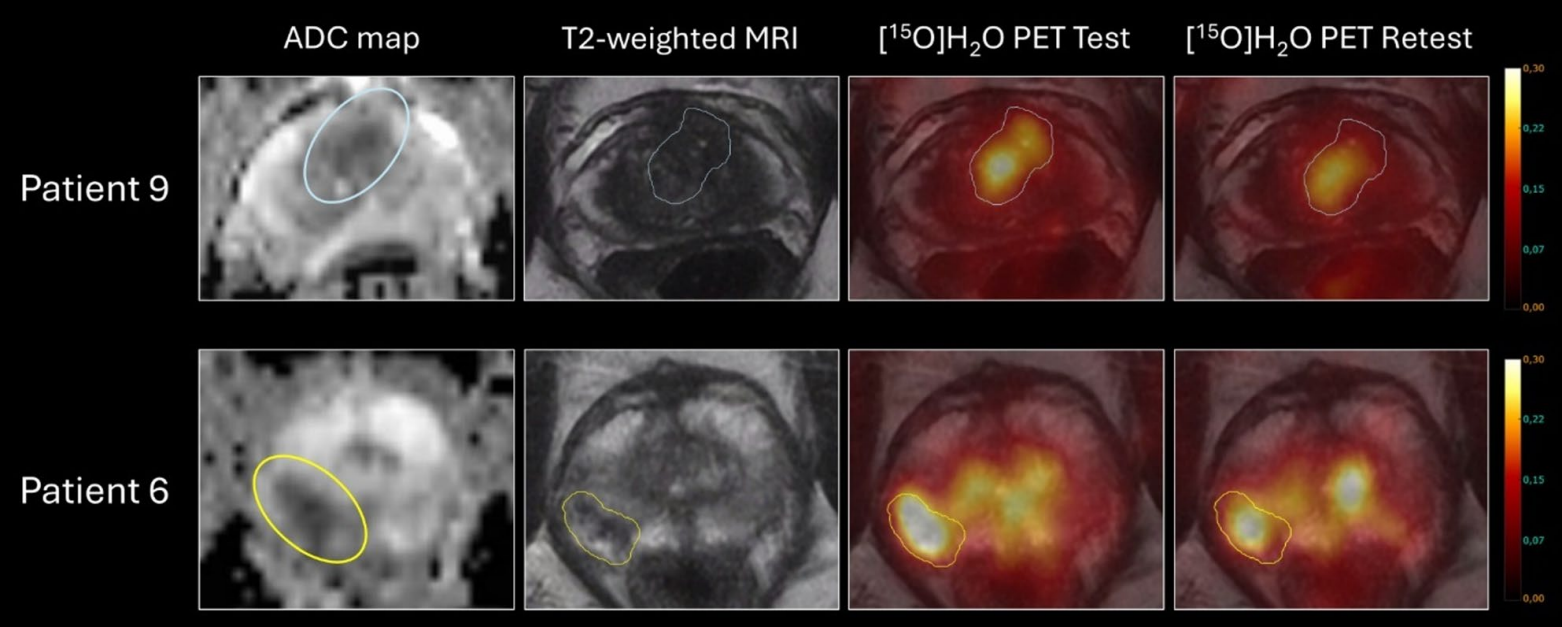
Test and retest scan images of two patients in the study. First row is patient nine with a PI-RADS 4 lesion involving both transitional and peripheral zone and second row is patient six with a PI-RADS 5 peripheral zone lesion. Both had tumours with high perfusion and significant prostate cancer, patient nine (first row) had biopsy ISUP Grade 3 and postprostatectomy ISUP Grade 5 and patient six (second row) had both biopsy and postprostatectomy ISUP Grade 2

**Fig. 2:**
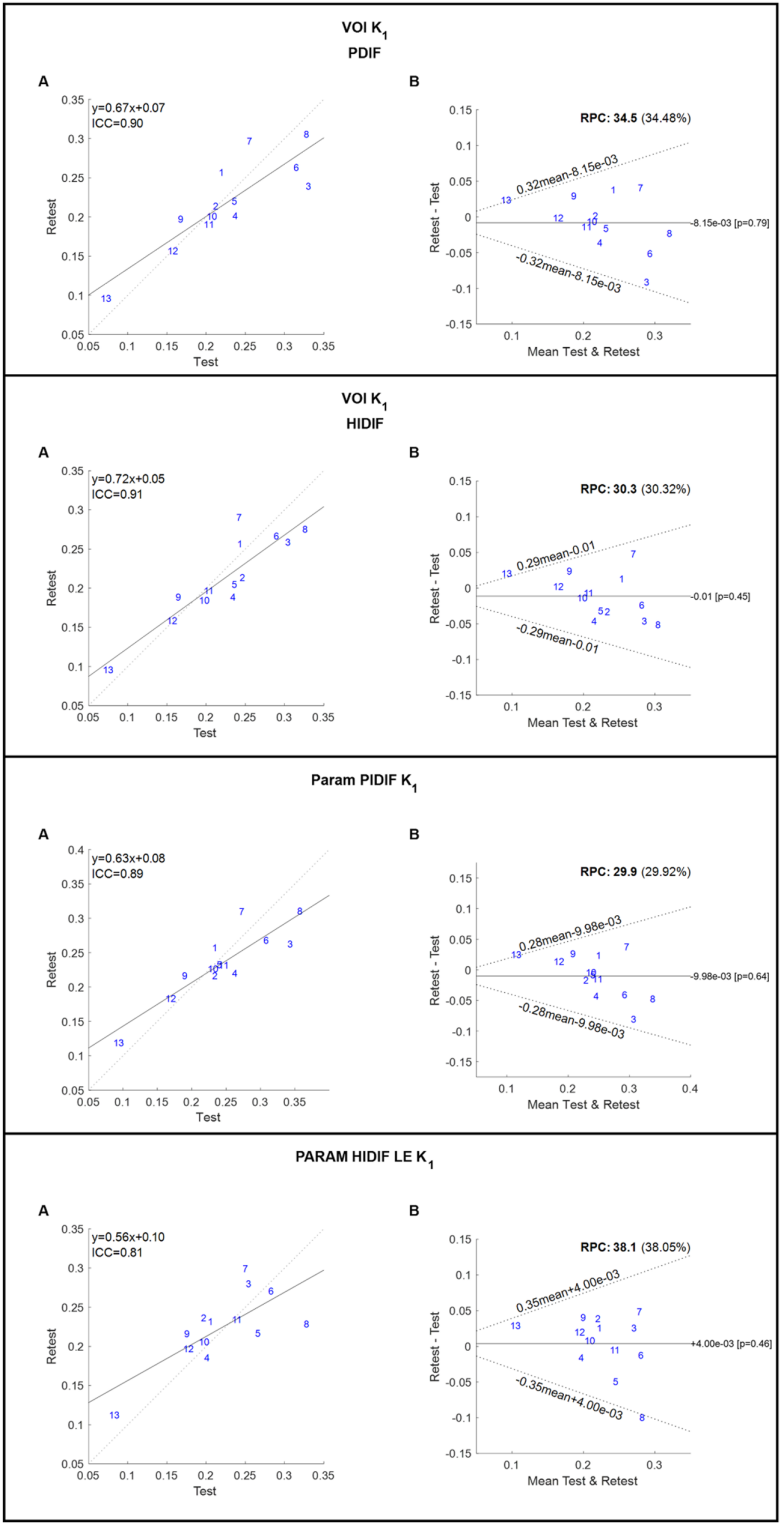
Repeated K_1_ measures are plotted against each other for VOI_PIDIF, VOI_HIDIF, Param_PIDIF and Param_LE (abbreviations explained in Table 1). Grey dashed line represents y = x, whereas solid black line is the linear fit. Linear equations and ICC are shown. Bland Altman plots for K_1_ for VOI_PIDIF, VOI_HIDIF, Param_PIDIF and Param_LE. Black solid line is mean difference between measurement 2 and 1. Black dotted lines are 95% upper and lower 95% limits of agreement (1.96 x sd). ICC: intraclass correlation. RPC: repeatability coefficient

In general, the different modelling approaches performed almost equally and correlated excellently both between VOI methods and between VOI and parametric methods. There was a tendency towards larger variation when applying the correction for dispersion (VOI_PIDIF) for the VOI method and when using the Leading-Edge method for delay correction in the parametric images (Param_LE) (Figs. 1 and 2).

Measures of ICC and repeatability coefficients are listed in Table 4. Repeatability for k_2_ was poor (repeatability coefficient 67.9% – 71.4%), probably explained by poor signal to noise ratio in small lesions with relatively low perfusion (Supplementary Fig. 1).

Sample size calculations for a longitudinal study on change in tumour perfusion based on the repeatability from the present study are found in Table 5.

**Table 4:** ICC and repeatability coefficient for K_1_ for the different modelling approaches

Measure	ICC	Repeatability (%)
VOI_PIDIF	0.90	34.48
VOI_HIDIF	0.91	30.32
Param_PIDIF	0.89	29.92
Param_LE	0.81	38.05

**Table 5:** Sample size calculations for longitudinal study on change in tumour perfusion based on the repeatability from the present study

	K_1_ (VOI PIDIF)	K_1_ (VOI HIDIF)	K_1_ (Param_PIDIF	K_1_ (Param_LE)
Sample Size (N) at −20% change	10	8	8	11
Sample Size (N) at −30% change	6	5	5	6
Sample Size (N) at −50% change	4	3	3	4

Sample size needed to detect relative changes of −20%, −30%, and − 50% were calculated using a 2-sided significance test for paired data with a significance level of 5% and a power of 95%

## Discussion

The main result of the present study is that tumour perfusion in primary prostate cancer can be measured with a repeatability of around 30%, meaning that increase or decrease above 30% in the individual patient is likely to represent actual changes in tumour physiology.

In contrast to our previous repeatability study on ^82^Rb PET, this study assessed the repeatability of the method unaffected by the day-to-day variability, which will inevitably affect longitudinal clinical scans during treatment. The patients were scanned on PET/MRI in the present study and instructed not to move between scans to ensure optimal alignment between MRI-derived VOIs and perfusion images. Besides, a thorough visual assessment of misalignment between T2 weighted MRI and parametric [^15^O]H_2_O PET K_1_ images was performed. Nevertheless, any movement between scans, and the misalignment it causes, will continue to pose a potential risk to the repeatability of the measurements. The present cohort of 10 patients with 13 primary lesions is comparable with most previous repeatability studies on PET tumour perfusion [15, 16, 21], and half the size of the cohort examined by Lodge et al. [14].

We tested four different modelling approaches (Table 1) and found comparable repeatability between the parametric and VOI-based methods. The voxel-wise delay correction applied in the parametric images showed poorer repeatability than the PIDIF-based approach. In contrast, for the VOI methods, incorporating dispersion correction (PIDIF) had only a minor impact on repeatability compared to using the pure HIDIF. Accordingly, parametric images using a delay- and dispersion-corrected IDIF (Param_PIDIF) as well as any of the VOI-based methods constitute robust approaches to tumour perfusion quantification. The repeatability of 30% found in the present study is comparable with those from Lodge et al. [14] and our previous study on ^82^Rb PET [16], while the studies from de Langen et al. [21] and van der Veldt et al. [15] found somewhat lower repeatability of 16–18%. Explanations for this deviation could be the method of input function and that we included much smaller tumours than previous studies, even below 1 cm^3^.

Lodge et al. discussed the possibility of reducing the repeatability to approximately 26% by averaging two repeated measurements at each time point [14]. Another possibility to increase repeatability could be to utilise the superior sensitivity of long axial field-of-view PET scanners, which would also obviate the need for a separate heart scan. However, the repeatability of [^15^O]H_2_O tumour perfusion on long axial field-of-view PET scanners remains to be determined.

Regarding primary prostate tumours, benign hyperplastic nodules with increased perfusion is one of the main challenges as shown in Fig. 1, second row. This illustrates both that PET perfusion is merely suited for tumour characterisation than for tumour detection and the importance of a separate modality for VOI definition.

Using PET/MRI has the obvious advantage of localising the tumour anatomically, which is needed for VOI definition, especially in subjects with concurrent benign hyperplasia.

## Future perspectives

As gold standard for non-invasive measurement of tumour perfusion, [^15^O]H_2_O PET is a robust tool for assessing tumour biology. With increasing availability of [^15^O]H_2_O generator systems and other perfusion agents, routine PET-assessment of quantitative tumour blood flow is now within reach at multiple PET-centres worldwide.

As tumour perfusion correlated to ISUP grade and hence aggressiveness in previous publications from our group, it is a relevant measure in prostate cancer [2–5]. Tumour perfusion is a marker of nutrient agnostic growth potential; hence it might represent a more relevant aspect of tumour biology compared to prostate-specific membrane antigen (PSMA) expression. FDG is not an obvious alternative as prostate cancer is often not FDG-avid.

Potential clinical applications could be for monitoring small tumours with low ISUP grade followed by urologists in active surveillance, since a more aggressive treatment approach might be considered for tumours with high K_1_. Besides, repeated tumour perfusion measurement might be a good method for monitoring the effect of local treatment of prostate cancer or for assessing effects of systemic neoadjuvant therapy prior to local treatment.

As these potential indications usually concern small tumours, the results from the present study are crucial, showing that tumour perfusion can be measured with high repeatability even in small tumours below 1 cm^3^.

Monitoring of oncological treatment response in patients with metastatic prostate cancer could potentially be another promising application.

Tumour hypoxia is associated with poor response to radiotherapy and worse patient outcome [22, 23]. Since tumour perfusion is critical for oxygen delivery to cancer cells, it plays a central role in the avoidance of tumour hypoxia. However, sufficient tumour perfusion does not guarantee absence of hypoxia, perhaps due to competing factors such as decreased diffusion capacity, increased oxygen consumption, heterogeneous microcirculation or chaotic tumour microvasculature [24, 25]. For PET hypoxia imaging, determination of the correlating tumour perfusion is important as low hypoxia PET signal can also be caused by flow-limited tracer delivery [24, 25]. Therefore, integrating hypoxia and perfusion metrics could provide a more comprehensive basis for disease characterisation [24, 26–28]. Furthermore, tumour perfusion is a key determinant of how effectively anti-cancer therapies are delivered to the tumour cells [29, 30].

Considering the aforementioned factors, perfusion PET has the potential to support both the planning and monitoring of a range of oncological therapies. PSMA radioligand therapy could be of interest, as the treatment effect could potentially be affected by both flow-based delivery to the tumour and perfusion-hypoxia related radiotherapy resistance.

The association between tumour heterogeneity and resistance to targeted precision therapy is well-established, as overall patient outcome are often driven by the lesion with the poorest response [31]. Therefore, it is crucial to capture and assess the biological response in all cancer lesions simultaneously during response monitoring.

With the introduction of long axial field-of-view PET scanners, quantitative assessment of tumour perfusion in patients with metastatic disease has become feasible, even with creation of parametric perfusion images [18, 32–35]. On this topic, the repeatability of [^15^O]H_2_O perfusion measurements in prostate cancer metastases is unknown.

The potential clinical applications of PET tumour perfusion imaging for characterisation and monitoring prostate cancer patients needs to be explored in future studies, which can be designed using the sample size calculations shown in Table 5.

## Conclusions

[^15^O]H_2_O perfusion PET is a repeatable method for measurement of tumour perfusion in localised prostate cancer, showing comparable performance when using parametric K_1_ perfusion maps and VOI-based analysis. For longitudinal evaluation, changes above 30% are likely to represent actual changes in tumour perfusion, for example as treatment response.

## Supplementary Information

Supplementary Material 1: Repeated k2 measures are plotted against each other for VOI_PIDIF and VOI_HIDIF. Grey dashed line represents y = x, whereas solid black line is the linear fit. Linear equations and ICC are shown. Bland Altman plots for k2 for VOI_PIDIF and VOI_HIDIF. Black solid line is mean difference between measurement 2 and 1. Black dotted lines are 95% upper and lower 95% limits of agreement. ICC: intraclass correlation. RPC: repeatability coefficient

## Data Availability

The datasets used in the current study are available from the corresponding author on reasonable request.

## Abbreviations

HIDIF: Heart image-derived input functions
ICC: Intraclass correlation coefficient
ISUP: International Society of Urological Pathology
LE: Leading edge
MRI: Magnetic resonance imaging
OSEM: Ordered subset expectation maximisation
PET: Positron emission tomography
PIDIF: Input function from pelvic arteries
PSF: Point-spread-function
TAC: Time-activity-curve
TOF: Time-of-flight
VOI: Volume of interest
