# Supplementary figures and images for: Repeatability of tumour perfusion measurement with [15O]H2O PET in prostate cancer
Source: EJNMMI Res. 2026 Jan 8;16:23. doi: 10.1186/s13550-026-01375-2 (PMC12881236; doi:10.1186/s13550-026-01375-2)

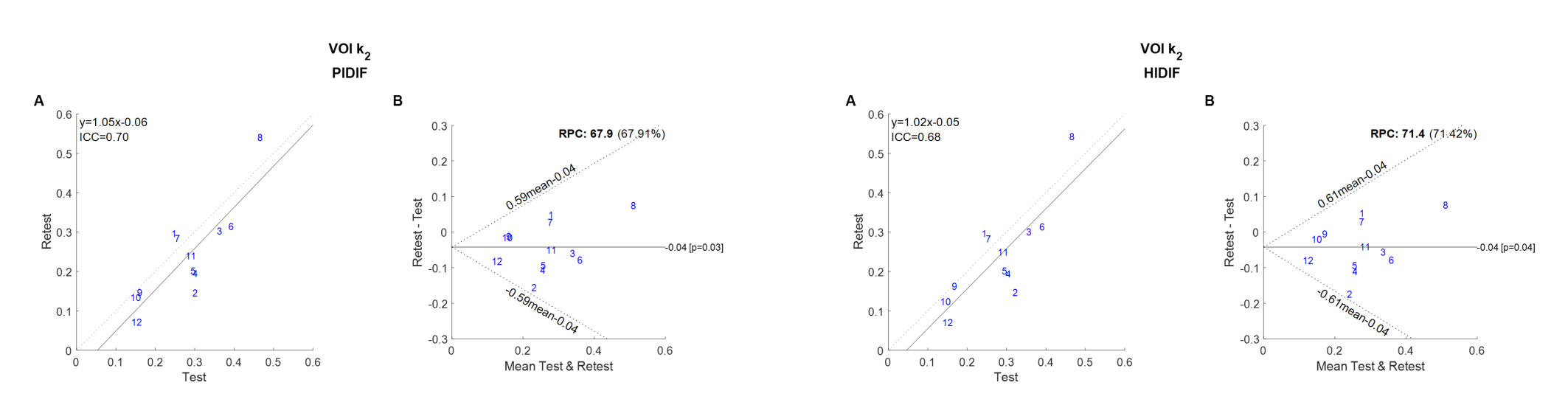

Supplement: Supplementary file 1 — Supplementary Material 1: Repeated k2 measures are plotted against each other for VOI_PIDIF and VOI_HIDIF. Grey dashed line represents y = x, whereas solid black line is the linear fit. Linear equations and ICC are shown. Bland Altman plots for k2 for VOI_PIDIF and VOI_HIDIF. Black solid line is mean difference between measurement 2 and 1. Black dotted lines are 95% upper and lower 95% limits of agreement. ICC: intraclass correlation. RPC: repeatability coefficient [file 13550_2026_1375_MOESM1_ESM.tif]
